# Nanostructured Lipid Carriers Made of Ω-3 Polyunsaturated Fatty Acids: In Vitro Evaluation of Emerging Nanocarriers to Treat Neurodegenerative Diseases

**DOI:** 10.3390/pharmaceutics12100928

**Published:** 2020-09-29

**Authors:** Sara Hernando, Enara Herran, Rosa Maria Hernandez, Manoli Igartua

**Affiliations:** 1 NanoBioCel Research Group, Laboratory of Pharmaceutics, School of Pharmacy University of the Basque Country (UPV/EHU), 01006 Vitoria-Gasteiz, Spain; sara.hernando@ehu.eus; 2 Biomedical Research Networking Centre in Bioengineering, Biomaterials and Nanomedicine (CIBER-BBN), Institute of Health Carlos III, 28029 Madrid, Spain; 3 Bioaraba, NanoBioCel Research Group, 01006 Vitoria-Gasteiz, Spain; 4 Biokeralty Research Institute, C/Albert Einstein 25 bajo, Edificio E-3 Miñano, 01510 Álava, Spain; enara.herran@keralty.com

**Keywords:** nanostructured lipid carriers, nanocarrier, docohexaenoic acid, neuroprotection, neuroinflammation

## Abstract

Neurodegenerative diseases (ND) are one of the main problems of public health systems in the 21st century. The rise of nanotechnology-based drug delivery systems (DDS) has become in an emerging approach to target and treat these disorders related to the central nervous system (CNS). Among others, the use of nanostructured lipid carriers (NLCs) has increased in the last few years. Up to today, most of the developed NLCs have been made of a mixture of solid and liquid lipids without any active role in preventing or treating diseases. In this study, we successfully developed NLCs made of a functional lipid, such as the hydroxylated derivate of docohexaenoic acid (DHAH), named DHAH-NLCs. The newly developed nanocarriers were around 100 nm in size, with a polydispersity index (PDI) value of <0.3, and they exhibited positive zeta potential due to the successful chitosan (CS) and TAT coating. DHAH-NLCs were shown to be safe in both dopaminergic and microglia primary cell cultures. Moreover, they exhibited neuroprotective effects in dopaminergic neuron cell cultures after exposition to 6-hydroxydopamine hydrochloride (6-OHDA) neurotoxin and decreased the proinflammatory cytokine levels in microglia primary cell cultures after lipopolysaccharide (LPS) stimuli. The levels of the three tested cytokines, IL-6, IL-1β and TNF-α were decreased almost to control levels after the treatment with DHAH-NLCs. Taken together, these data suggest the suitability of DHAH-NLCs to attaining enhanced and synergistic effects for the treatment of NDs.

## 1. Introduction

Neurodegenerative diseases (ND) cause progressive loss of brain functions and overlapping clinical syndromes. Among the many risk factors associated with neurodegeneration, the aging process itself has by far the most impact. However, other environmental and genetic aspects are associated with the risk of suffering these diseases. Indeed, the NDs are the result of a combination of different environmental risk factors, such as vascular risk, tobacco consumption, alcohol and aging, together with different genes involved in the development of these central nervous system (CNS) disorders. For example, in Alzheimer’s disease (AD), APOE4 (apolipoprotein E) is the major genetic risk factor. Moreover, mutations in APP, (amyloid precursor protein) PSEN1 (presenilin proteins) and PSEN2 probably accelerate the toxic accumulation of proteins that leads to the undergoing neurodegenerative process present in AD. Another example is mutation in GBA (glucocerebrosidase), which encodes β-glucocerebrosidase. Alterations in this gene lead to lysosomal enzyme deficiency and an increase in the prevalence of Parkinson diseases (PD). Other mutations in genes, such as LRRK2 (leucine-rich repeat kinase 2), parkin and SNCA, (alpha synuclein) which encodes the protein α-synuclein, are the most common causes of dominantly and recessively inherited PD. These NDs are age-dependent disorders that are becoming increasingly prevalent as life expectancy rises worldwide. This prevalence will increase, becoming a serious economic burden and public health problem. Despite AD and PD being the most common NDs, Huntington’s disease (HD), amyotrophic lateral sclerosis (ALS), frontotemporal dementia and the spinocerebellar ataxias are also examples of NDs, with aging as the main risk factor for all of them [1,2,3]. Although they have different clinical manifestations and symptoms, they share common features and mechanisms of neurodegeneration, highlighting protein deposits, mitochondrial homeostasis, stress granules and synaptic toxicity, together with a maladaptive innate immune response that converges in the form of chronic inflammation characterized by reactive gliosis and an increase in proinflammatory cytokines [4,5].

Despite the significant public health issues NDs have, to date, the treatments for these diseases remain symptomatic without halting the progression of the disease. Moreover, the lack of an overall positive effect on the clinical manifestation of the disease, together with the presence of systemic side effects, has prompted the patients to abandon their therapies [6,7]. Due to the lack of an effective treatment for NDs, in the last few years, promising new molecules, such as neurotrophic factors (NTFs), antioxidant molecules and polyunsaturated fatty acids (PUFAs), have been raised as a feasible therapeutic options to target the undergoing oxidative stress and inflammatory status or to enhance neurogenesis [8,9,10].

Nevertheless, no matter the treatment, one of the most challenging obstacles for an effective therapy for NDs is the low penetration efficiency of drugs to the CNS due to the presence of the blood brain barrier (BBB). In the last few years, different strategies have been developed in order to achieve brain targeting. These strategies include direct and indirect methods. The direct or invasive techniques include surgical methods to administer drugs directly into the brain and the disruption of the BBB to open it. Meanwhile, the indirect or noninvasive techniques include nonaggressive approaches to access the brain without affecting this barrier integrity [11,12]. These noninvasive techniques include alternative systemic administration routes like intranasal administration [13,14]. Gartziandia et al. conducted a study that successfully showed the brain delivery of therapeutics after intranasal administration with lipid nanoparticles coated with chitosan (CS) [15]. Moreover, the combination of the formulation with cell-penetrating peptides (CPP) has been disclosed as a useful strategy to enter the brain [16,17]. Anyway, one of the most studied approaches to attain this goal are nanotechnology-based drug delivery systems [18,19]. Among them, nanoparticles (NPs) have been widely used as a promising approach for ND treatment. NPs are highly stable 3D encapsulation systems that can be loaded with drugs and functionalized with targeting ligands or antibodies, and they can be used as nanocarriers to deliver drugs to the CNS [20]. Among other materials, natural or synthetic polymers and lipids have been employed in NP development [11].

Indeed, numerous research papers have combined NPs with well-known treatments or therapeutic approaches that have recently appeared, such as growth factors (GFs), antioxidant molecules and PUFAs entrapped in the nanoformulation [21,22,23,24]. All these research papers support the use of nanoparticles, offering many advantages over traditional formulations, such as protecting the molecule from degradation, increasing the half-life of the therapeutic molecules and, therefore, limiting multiple dosing and decreasing side effects. Among others, the nanostructured lipid carriers (NLCs) are an unstructured solid lipid matrix made of a mixture of blended solid and liquid lipids and an aqueous phase with a mixture of surfactants [25]. In addition, they have gained the attention of researchers since they exhibit a lack of toxicity, high drug loading capacity of both hydrophobic and hydrophilic compounds and a natural tendency to pass across the BBB [26]. Moreover, the NLCs can be functionalized with different substances to increase their tendency to pass through the BBB [27]. Examples of these molecules are chitosan (CS) and the cationic cell-penetrating peptide TAT; both molecules have increased brain targeting of therapeutic molecules for NDs treatment, as we pointed out in previous publications [15,24].

Up to today, most of the lipids used for NLC formulation are inert excipients, without any active role in preventing or treating diseases [28]. Indeed, only a few research groups have described the use of functional lipids that could play a therapeutic role in forming the lipid matrix, i.e., Ω-9 oleic acid incorporated into a NLC formulation for dermal applications [29]. Other kinds of functional lipids are PUFAs, Ω-3 and Ω-6 polyunsaturated fatty acids. As previously pointed out, PUFAs have been raised as a promising new approach to target neurodegenerative diseases. Although the biochemical mechanism undergoing the beneficial effect in NDs is not clear at all, they have exhibited the positive effect of decreasing the neuroinflammation process undergoing NDs, improving memory in animal models of AD, sensory motor tests in PD animal models and inhibiting amyloid-β fibrils both in vitro and in vivo [30,31,32]. Such functional lipids, which are also called nutraceutical, have proved to be a useful tool to manage NDs [33]; although they cannot totally restore brain functions, they may be beneficial to manage some symptoms of the disease or just as a coadjutant treatment. Actually, in the last few years, numerous observational studies showed an association between a diet rich in PUFAs and a lower risk of PD. Moreover, dietary treatments with PUFAs have shown to decrease the inflammatory status of these NDs and decrease depressive symptoms, among others [34,35,36].

Therefore, taking into account the promising results obtained with PUFAs in ND treatment, along with the beneficial effects of NLCs modified with CS and TAT for brain targeting, the objective of this research article is to combine both strategies for ND treatment. More concretely, the goal of the present work is to develop NLC with a functional lipid, such as DHA (docohexaenoic acid) and its hydroxylated derivate (DHAH), so the nanoparticles themselves could exhibit neuroprotective and antiinflammatory effects. In summary, we aim to demonstrate the beneficial effect of PUFAs incorporated to the NLC matrix, generating a new functional nanocarrier for entrapping different therapeutic molecules in the future and acting as a synergetic therapy.

## 2. Materials and Methods

### 2.1. Materials

Precirol ATO 5^®^ (glycerol disterate) (pharma grade) and Mygliol^®^ (caprylic/capric triglyceride) (pharma grade) were a kind gift from Gattefosé (Lyon, France) and IOI Oleo GmbH, respectively. DHA (80% purity) and DHAH (85.4% purity) fatty acids in ethyl ester and triglyceride form were purchased from Medalchemy (Alicante, Spain). They were aliquoted in topaz vials in N_2_ inert atmosphere conditions ready for one unique use in order to avoid oxidation during storage over the course of the experiment. Tween 80, Tween 20 and 3.7% paraformaldehyde were purchased from Panreac (Barcelona, Spain). Lutrol^®^ F-68 (Poloxamer 188) (FDA-approved excipient) was acquired from VWR Avantor (Barcelona, Spain). Chitosan (CS) was obtained from NovaMatrix (Sandvika, Norway). Trehalose dehydrate, Triton X-110, bovine serum albumin (BSA), poly-l-lysine hydrobromide (PLL), deoxyribonuclease I from a bovine pancreas (DNase I), 6-hydroxydopamine hydrochloride (6-OHDA), Cell Counting Kit-8 (CCK-8), 2,2′-Azino-bis(3-ethylbenzthiazoline-6-sulfonic acid) (ABTS) and Fluoromount^TM^ Aqueous Mounting Medium were acquired from Millipore Sigma Life Sciences (Madrid, Spain). Additionally, 4′,6-diamidino-2-phenylindole (DAPI), donkey anti-mouse IgG (H+L) cross-adsorbed secondary antibody, Alexa Fluor 555, goat anti-rabbit IgG (H+L) cross-adsorbed secondary antibody and Alexa Fluor 488 were purchased from Thermo Fisher Scientific (Madrid, Spain). Neurobasal^TM^ medium, B-27^TM^ supplement, glutamine 100X, penicillin-streptomycin (P/S), fetal bovine serum (FBS), DMEM GlutaMAX^TM^, trypsin-EDTA 0.25% and Hank’s Balanced Salt Solution (HBSS) were obtained from Gibco© by Life Technologies (Madrid, Spain). Anti-tyrosine-hydroxilase (TH) primary antibody and anti-glial fibrillary acidic protein (GFAP) primary antibodies were obtained from Abcam (Cambridge, UK). Anti-Iba1 (ionizing calcium-binding adaptor molecule 1) primary antibody was purchased from Synaptic Systems (Göttingen, Germany). Lipopolysaccharide (LPS) suitable for cell culture was bought from InvivoGen (Toulouse, France). Rat IL-6 Standard, Rat IL-1β Standard and Rat TNF-α Standard ABTS ELISA Development Kits were obtained from Peprotech (London, UK). Finally, TAT (>95% purity) was obtained from ChinaPeptides (Suzhou, China). All reagents used were of analytical grade.

### 2.2. NLC Preparation and Optimization

NLC were prepared based on a previously described melt-emulsification technique [16,24]. Four different formulations were developed, combining solid and liquid lipids. The solid lipid Precirol ATO 5^®^ (melting point: 56 °C) was used in all formulations; however, different liquid lipids in a different solid:lipid ratio were used, as shown Table 1. All nanoformulations shown in Table 1 were performed in triplicate.

DHA, its hydroxylated derivate in ethyl ester form (DHAH-EE) and Mygliol^®^ (caprylic/capric glyceride) were liquid at room temperature; however, the hydroxylated derivate of DHA in triglyceride form (DHAH-TG) was slightly more viscous. We used two different variants of DHAH, its hydroxylated derivate in ethyl ester form (DHAH-EE) and triglyceride form (DHAH-TG) in order to select the most suitable one to develop our new nanocarrier.

The lipid phase, containing both the solid and the liquid lipid, was heated 5 °C above its melting point until a clear and homogeneous phase was obtained. The lipid phase was formed with a different solid:liquid lipid ratio, as described in Table 1. The aqueous solution was composed of Tween 80 (3% *w*/*v*) and poloxamer 188 (2% *w*/*v*) for a final volume of 4 ml. The aqueous phase was warmed and added to the melted oily phase and sonicated for 60 s at 50 W (Branson^®^ Sonifier 250, Fisher Scientific, Madrid, Spain). The obtained nanoemulsion was maintained under magnetic stirring for 15 min at room temperature and stored at 4 °C overnight to allow the recrystallisation of the lipid for NLC formation. On the following day, the nanoparticle dispersion was centrifuged in an Amicon filter (Amicon, “Ultracel-100k”, Millipore Sigma Life Sciences, Madrid, Spain) at 2500 rpm (MIXTASEL, JP Selecta, Barcelona, Spain) for 15 min and the nanoparticles were washed three times with milli Q water. Finally, 15% *w*/*w* trehalose was added as cryoprotectant, and the NLCs were lyophilized for 42 h (LyoBeta 15, Telstar, Spain).

Prior to the NLC coating process, TAT was covalently linked to CS by a surface activation method previously described by our research group [16] The CS: TAT employed ratio was 1:0.01 (*w*/*w*). TAT conjugation with CS was prepared through a carbodiimide-mediated coupling reaction [37]. The reaction was made though the EDC/NHS technique, in which the reactive sulfo-NHS ester reacted with the amine functionality of proteins and peptides via the formation of an amide bond [38,39]. Briefly, 250 μL EDC (1-ethyl-3-(3-dimethylaminopropyl) carbodiimide hydrochloride) in solution (1 mg/mL) and 250 μL of sulfo-NHS (*N*-hydroxysulfosuccinimide) in 0.02 M phosphate buffered saline (PBS) were added dropwise to a 4 mL CS solution (0.5% *w*/*v* in PBS 0.02 M) under magnetic stirring (2 h at room temperature). For the coupling of TAT, a 250 μL TAT solution (1 mg/mL) in PBS (0.02 M; pH 7.4) was added dropwise to the activated CS under gentle agitation. The TAT-CS solution was maintained under agitation for another 4 h at room temperature and then incubated at 4 °C overnight. The next day, the NLC were coated with TAT-CS; for that purpose, an NLC dispersion previously prepared was added dropwise to the TAT-CS solution under continuous agitation for 20 min at room temperature. After the coating process, CS-NLC-TAT nanoformulation was centrifuged in Amicon filters (Amicon, “Ultracel-100k”, MiIlipore, Millipore Sigma Life Sciences, Madrid, Spain) at 2500 rpm (MIXTASEL, JP Selecta, Barcelona, Spain) for 15 min and washed three times with Milli Q water. Finally, the nanoformulation was freeze-dried with the cryoprotectant trehalose at a final concentration of 15% (*w*/*w*) of the weighed lipid, and then it was lyophilized for 42 h (LyoBeta 15, Telstar, Terrassa, Spain). This final step, regarding the coating with CS and TAT, was only performed after the election of the lipid type and the solid:lipid ratio, resulting in the formulations named (CS-TAT) DHAH-NLC, or just DHAH-NLC, and (CS-TAT) Mygliol-NLC, or just Mygliol-NLC, used in the following described studies in primary cell cultures.

### 2.3. NLC Characterization

The mean particle size (Z-average diameter) and the polydispersity index (PDI) were measured by dynamic light scattering (DLS), and the zeta potential was determined through laser Doppler micro-electrophoresis (Malvern^®^ Zetasizer Nano ZS, Model Zen 3600; Malvern Instruments Ltd., Malvern, UK). Three independent measurements were performed for each formulation. The data are represented as the mean ± SD. Nanoparticle surface characteristics and morphology were examined by transmission electron microscopy (JEOL JEM 1400 Plus, Izasa Scientific, Madrid, Spain). The thermal behavior of the nanoparticles and the excipients and components themselves were studied using differential scanning calorimetry (DSC) (DSC-50, Shimadzu, Kioto, Japan). Each sample was sealed in an aluminum pan and heated from 25 °C to 350 °C at a heating rate of 10 °C per minute. The sample size was 1–2 mg for each measurement. Finally, Fourier transform infrared (FTIR) spectra of Mygliol-NLC and DHAH-NLC with and without CS and TAT coating were carried out on a Nicolet Nexus FTIR spectrometer using ATR Golden Gate (Thermo Scientific, Madrid, Spain) with a crystal ZnSe. The sample was placed directly onto the ATR crystal and the spectrum was obtained in transmittance mode. Each spectrum was the result of an average of 32 scans at 4 cm^−1^ resolution. Measurements were recorded in the wavelength range of 4000–750 cm^−1^.

### 2.4. Cell Cultures

#### 2.4.1. Primary Dopaminergic Cell Culture

Dopaminergic neuronal cultures were prepared from Wistar rat embryos at 15, 16 or 17 days of gestation (E15–E17). Animal procedures were reviewed and approved (3 April 2017) by the Local Ethical Committee for Animal Research of the University of the Basque Country (UPV/EHU, CEEA, ref. M20/2017/019). All of the experiments were performed in accordance with the European Community Council Directive on “The Protection of Animals Used for Scientific Purposes” (2010/63/EU) and with Spanish Law (RD 53/2013) for the care and use of laboratory animals.

**Figure 1:**
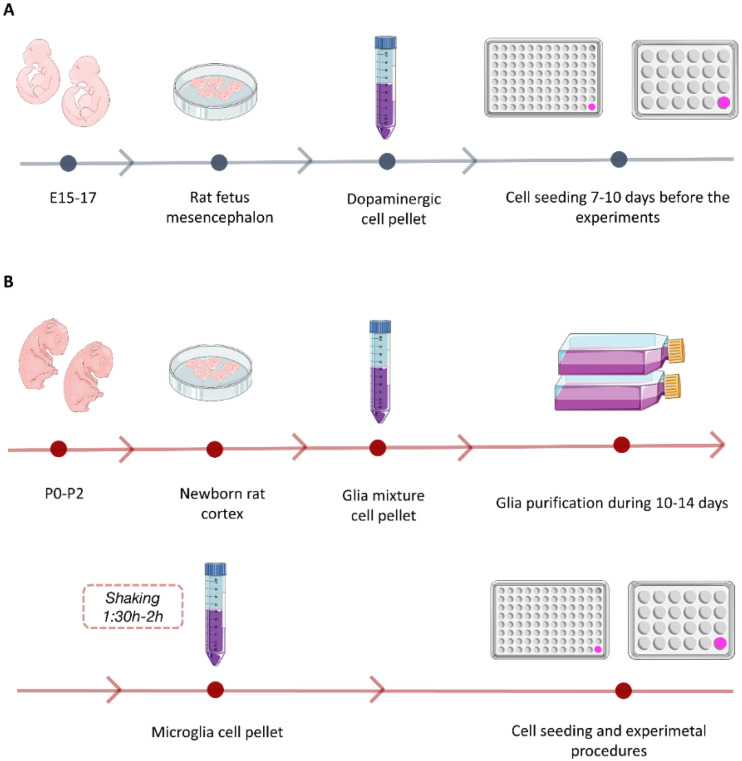
Schematic representation of primary cell culture protocols. (**A**) Primary dopaminergic neuron culture isolation and seeding. (**B**) Primary microglia cell culture isolation and seeding. This figure was created using Servier Medical Art templates, which are licensed under a Creative Commons Attribution 3.0 Unported License (https://smart.servier.com).

To obtain primary dopaminergic cell cultures, the following protocols were followed with slight modifications [40,41] (Figure 1A). Pregnant rats were euthanized and, under no aseptic conditions, the whole brain was removed from the rat embryos and kept on ice in a Petri dish (10 cm ∅) with HBSS for removing the blood vessels and meninges. Petri dishes containing embryos’ brains were put under the magnifying glass to select the brain areas of interest following Gaven et al. protocol [42]. The ventral portion of the mesencephalic flexure, a region of the developing brain rich in dopaminergic neurons, was used for cell preparations. After collecting the brain areas of interest, tissue was incubated with tryspin at 37 °C for 15 min under aseptic conditions. Then, it was also incubated with DNase for 30 s and, after that time, the trypsin was inactivated and the tissue was washed twice with DMEM GlutMAX^TM^ FBS 10%, P/S. Then, the tissue was mechanically dissociated by several passages through a 5 and 2 mL pipette. Finally, the cell suspension was passed through a 100 μm nylon strainer and centrifuged for obtaining a cell pellet. The cell pellet was resuspended in neurobasal medium supplemented with 0.5 mM glutamine, 1% antibiotic and 3% B27. Cells were seeded in 96-well culture plates (for the cell viability assay, neuroprotective activity assay and 6-OHDA toxin-induced assay) and in 24-well culture plates with glass coverslips (for the immunofluorescence assay), both previously coated with poly-l-lysine to promote cell adhesion, at a density of 40 × 10^3^ cells/well and 150 × 10^3^ cells/well, respectively.

#### 2.4.2. Primary Microglia Cell Culture

Microglia neuronal cultures were prepared from Wistar rat puppets at day 0 to 2 (P0–P2). Animal procedures were reviewed and approved by the Local Ethical Committee for Animal Research of the University of the Basque Country (UPV/EHU, CEEA, ref. M20/2017/035). All the experiments were performed in accordance with the European Community Council Directive on “The Protection of Animals Used for Scientific Purposes” (2010/63/EU) (22 September 2010) and with Spanish Law (RD 53/2013) (8 February 2013) for the care and use of laboratory animals.

Primary microglia cell cultures were obtained following Chen et al. protocol with slight modifications [43] (Figure 1B). Puppets’ brains were removed and kept on ice in a Petri dish (10 cm ∅) with HBSS under magnifying glasses for removing the meninges and selecting the brain area of interest. Then, the cortex of the brain was collected and incubated with trypsin for 15 min at 37 °C. The tissue was incubated with DNase for 30 s and, afterwards, the trypsin was inactivated and the tissue was washed twice with DMEM GlutMAX^TM^ FBS 10%, P/S. Finally, the tissue was mechanically dissociated by several passages through a 5 and 2 mL pipette and, then, passed through a 70 μm nylon strainer and centrifuged for obtaining a cell pellet. The obtained pellet was resuspended in DMEM GlutMAX^TM^ FBS 15%, P/S and incubated in a poly-l-lysine-coated flask at 37 °C in a humidified 5% CO_2_ atmosphere. After 3 days, full media were changed to DMEM GlutMAX^TM^ FBS 10%, P/S. After this, half media changes were done every 2–3 days for maintaining this glia mixture culture for 10–14 days. After that period, the flask media were removed and replaced with DMEM GlutMAX^TM^ FBS 15% P/S 24 h before microglia cell isolation started. Microglia cells were detached from the astroglia layer by shaking the flasks at 250 rpm for 1.5–2 h. Then, primary microglia cells were removed from the flask, resuspended in complete DMEM with FBS 15% and seeded in poly-l-lysine-precoated 96-well culture plates (for cell viability and cytokine release assays) and in 24-well culture plates with glass coverslips (for the immunofluorescence assay) at a density of 50 × 10^3^ cells/well and 150 × 10^3^ cells/well, respectively.

### 2.5. Immunofluorescence

Primary cells were seeded at a density of 150 × 10^3^ cells/well in 24-well culture plates with glass coverslips precoated with poly-l-lysine. At the time of interest, cells were fixed with 3.7% paraformaldehyde (PFA) for 10 min and, then, washed three times in PBS. Then, they were blocked and permeabilized with 1% (*w*/*v*) of BSA solution and 0.1% (*v*/*v*) Triton X-100 in PBS for 1 h at room temperature. After rinsing, they were incubated in rabbit polyclonal antibody Iba-1 (1:1000), mouse monoclonal anti-GFAP (1:2000) or rabbit polyclonal antibody TH (1:1000), respectively, with 1% (*w*/*v*) BSA and 0.1% Triton X-100 in PBS with agitation on at 4 °C. The following day, cells were incubated with the secondary antibody: anti-rabbit Alexa Fluor IgG 488 (1:1000) or anti-goat Alexa Fluor IgG 555 (1:1000), respectively, in PBS with 1% BSA and 0.1% Triton X-100 for 2 h at room temperature. After three rinsing steps, the fixed cells were incubated in DAPI (1:10,000) in PBS for 10 min. Then, they were washed twice with PBS and mounted with Fluoromount^TM^.

### 2.6. Cell Viability Assay

As previously pointed out, for cell viability study, the cells were seed in 40 × 10^3^ cells/well in poly-d-lysine-precoated 96-well culture plates. Dopaminergic cells were maintained for 7–10 days before performing the experiments and the medium was changed every 3 days, if necessary. At a determined time point, the media from the cells were removed and new fresh media were added with the different nanoparticle concentrations, DHAH-NLCs or Mygliol-NLCs, (100, 75, 50, 25, 12.5 and 5 μM) for 24 or 48 h. The concentration (μM) refers to the quantity of DHAH present in the NLC formulation. For dosing Mygliol NPs, an equal amount of NLCs was used as an internal control to observe the difference between using a functional lipid, such as DHAH, versus an inert lipid, such as Mygliol^®^.

Afterwards, the viability was assessed using the CCK-8 kit. Briefly, 10 μL of the CCK-8 reagent was added to the cells. After 4 h of incubation, the absorbance of the mixture was read at 450 nm, using 650 nm as the reference wavelength (Plate Reader Infinite M200, Tecan, Männedorf, Switzerland). The absorbance was directly proportional to the number of living cells in the culture. Cell viability for each condition is represented in a percentage related to the control positive (C^+^), where no treatment was added to the cell media.

In the case of microglia, slight modifications were done in the cell viability assay protocol. After obtaining a pure microglial cell culture, as previously pointed out, the microglia cells were seeded in 50 × 10^3^ cells/well density. Some 24 h after cell attachment, the media were removed and the cells were incubated for 24 or 48 h with the different concentrations of NLCs, as previously pointed out. Then, the viability CCK-8 test was performed as earlier described.

### 2.7. 6-OHDA Toxicity Assay

After 7–10 days of maintaining dopaminergic culture, the media were removed and different concentrations of 6-OHDA toxin were added (500, 100, 50, 25, 10 and 5 μM) for 24 h to correlate neurotoxin concentration to cell viability; for C^+^, we just changed the media and, for control negative (C^−^), DMSO 10% was added. To assess cell viability, after 24 h of incubation with 6-OHDA neurotoxin, the media were removed and the cells were fixed with 3.7% paraformaldehyde (PFA) for 10 min and, then, washed three times in PBS. DAPI staining (1:10,000) in PBS for 15 min was used to determine viable cells. For cell viability quantification, fluorescence microscopy images were obtained by means of an inverted microscope (Nikon TMS, Hampton, NH). Two images per well and three wells were used for each group; in total, six images were taken per group. Dopaminergic cells were scored as positive if they exhibited defined nuclear counterstaining. The data are expressed as the percentage of the C^+^ group with no treatment, which was set as 100%.

### 2.8. Neuroprotective Assay

**Figure 2:**
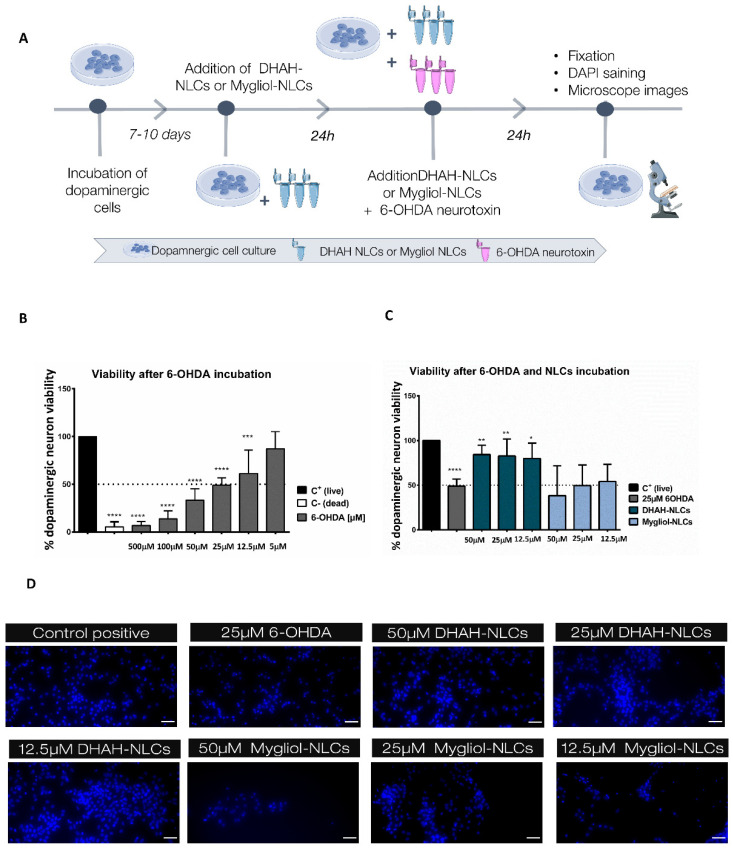
(**A**) Schematic representation of dopaminergic cell-based assays to evaluate the neuroprotective effects of DHAH-NLCs and Mygliol-NLCs. This figure was created using Servier Medical Art templates, which are licensed under a Creative Commons Attribution 3.0 Unported License (https://smart.servier.com). (**B**) Graphic representation of dopaminergic neuron viability after 24 h of incubation with different doses of 6-OHDA (**** *p* < 0.0001 C^+^ vs C^−^, 500 μM, 100 μM and 25 μM *** *p* < 0.001 C^+^ vs 10 μM); one-way ANOVA. (**C**) Graphic representation of the neuroprotective effect of DHAH-NLCs (** *p* < 0.01 25 μM 6-OHDA vs 50 μM DHAH-NLCs and 25 μM DHAH-NLCs, * *p* < 0.05 25 μM 6-OHDA vs 12.5 μM DHAH-NLCs); one-way ANOVA. (**D**) Representative fluorescence images of the neuroprotective assay with DAPI staining. The scale bar indicates 50 μM.

After 7–10 days of maintaining a dopaminergic culture, a neuroprotective assay was carried out with the developed nanocarriers (Figure 2A). The media were removed and different concentrations of DHAH-NLCs and Mygliol NLCs were added to the cells (50, 25 and 12.5 μM) 24 h before the 6-OHDA neurotoxin was added to the culture. 24 h after, the media were removed and fresh media were added, with a final concentration of 25 μM 6-OHDA neurotoxin and the previously tested concentrations for NLCs (50, 25 and 12.5 μM). To assess the neuroprotective effect of DHAH-NLCs against 6-OHDA neurotoxin, the media were removed 24 h later and cells were fixed with 3.7% paraformaldehyde (PFA) for 10 min and, then, washed three times in PBS. DAPI staining (1:10,000) in PBS for 15 min was used to determine viable cells. For cell viability quantification, fluorescence microscopy images were obtained by means of an inverted microscope (Nikon TMS, Hampton, NH). Two images per well and three wells were used for each group; in total, six images were taken per group. Dopaminergic cells were scored as positive if they exhibited defined nuclear counterstaining. The data are expressed as the percentage of the C^+^ group with no treatment but just media change, which was set as 100%.

### 2.9. Proinflammatory Cytokine Release Quantification: TNF-α, IL-1β and IL-6

**Figure 3:**
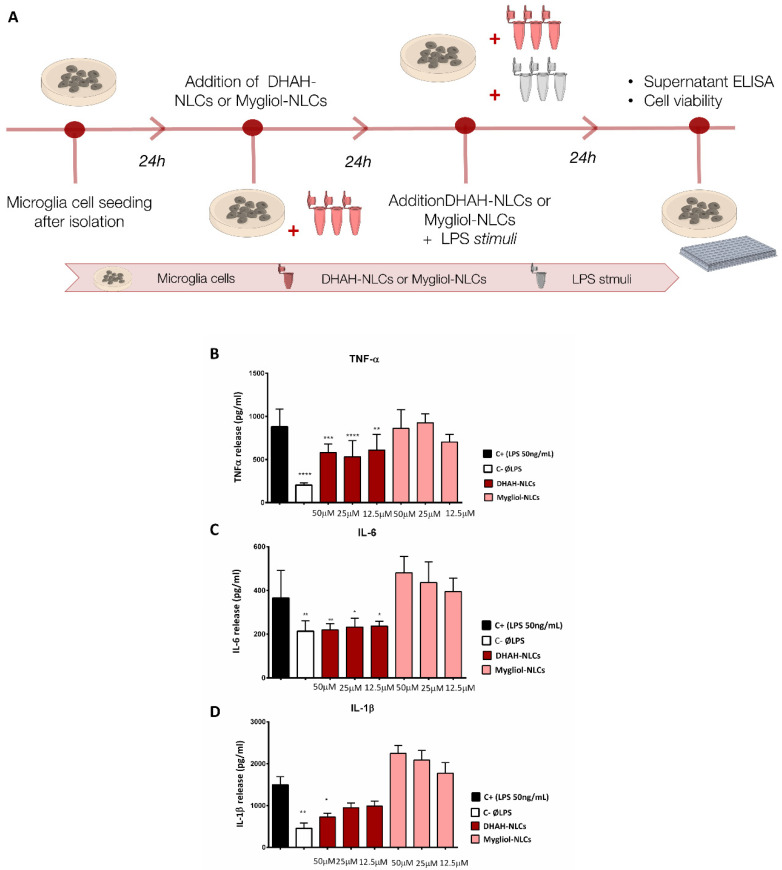
(**A**) Schematic representation of the antiinflammatory assay with DHAH-NLCs and Mygliol-NLCs. This figure was created using Servier Medical Art templates, which are licensed under a Creative Commons Attribution 3.0 Unported License (https://smart.servier.com). (**B**) Graphic representation of TNF-α values (pg/mL) for all the different tested concentrations and formulations (**** *p* < 0.0001 C^+^ vs. C^−^ and 25 μM DHAH-NLCs, *** *p* < 0.001 C^+^ vs. 50 μM DHAH-NLCs, ** *p* < 0.01 C^+^ vs. 12.5 μM DHAH-NLCs); one-way ANOVA. (**C**) Graphic representation of IL-6 values (pg/mL) for all the different tested concentrations and formulations (** *p* < 0.01 C^+^ vs. C^−^ and 50 μM DHAH-NLCs, * *p* < 0.05 C^+^ vs. 25μM DHAH-NLCs and 12.5μM DHAH-NLCs); one-way ANOVA. (**D**) Graphic representation of IL-1β values (pg/mL) for all the different tested concentrations and formulations (** *p* < 0.01 C^+^ vs. C^−^ and * *p* < 0.05 C^+^ vs. 5 μM DHAH-NLCs); one-way ANOVA.

DHAH-NLCs antiinflammatory effect against LPS (lipopolysaccharide) was carried out in microglia primary cells (Figure 3A). To perform the assay, cells were pretreated for 24 h with DHAH-NLCs and Mygliol-NLCs at different concentrations selected from studies previously carried out in Section 2.6 (50 μM, 25 μM and 12.5 μM) or just media change. After that treatment, media were removed and cells were incubated for another 24 h with LPS 50 ng/mL and the different concentration of the nanoparticles. After that 24 h, the cell media supernatant was collected and stored at −80 °C. The levels of TNF-α, IL-1β and IL-6 were analyzed with ELISA assay (Peprotech, London, UK). The total amount of cytokine release was normalized according to cell viability measured with CCK-8 assay at the same time point.

### 2.10. Statistical Analysis

All results are expressed as mean ± SD. The results obtained from the cell culture have been performed in *n* = 3 biological replicates for all the experiments described in this article. Experimental data were analyzed using the computer program GraphPad Prism (v. 6.01, GraphPad Software, San Diego, CA, USA). One-way ANOVA was used for analyzing all the data represented in this research article. *p* values <0.05 were considered significant.

## 3. Results

### 3.1. Nanoparticle Characterization

The aim of the present work was to develop a new nanocarrier enriched in functional lipids, such as Ω-3 fatty acids, to form the lipid matrix of NLCs. For that purpose, we tried to substitute the lipids of the nanoformulation with different kinds of Ω-3 fatty acids. Precirol ATO 5^®^ was maintained as solid lipid. All modifications in the optimization process of the nanoformulation were performed in the liquid lipid, DHA, DHA-TG and DHA-EE, modifying the liquid lipid type and also the ratio of solid:lipid to formulate NLCs, since the main goal of this research was to increase the percentage of these functional lipids up to the maximum to form this new nanocarrier. In order to achieve this aim, we increased the percentage of lipid liquid from the 0.25% that we used in previous studies with Mygliol^®^ [44,45,46] to 1.25%, as described in Table 1. We used DHA and two different types of DHAH to select the most suitable one to prepare the NLCs, and we worked with Mygliol^®^, the liquid lipid previously used in our research group for different clinical applications [15,47], as our control.

**Figure 4:**
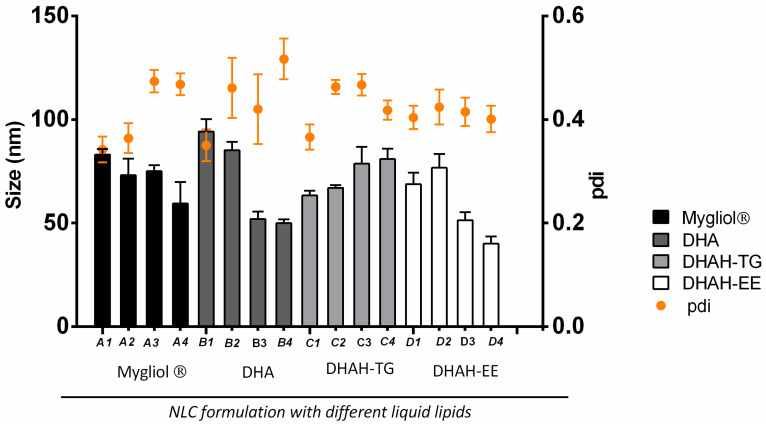
Characterization of the developed nanostructured lipids (NLCs) (*n* = 3 for all the nanoformulations).

Between DHAH-EE and DHAH-TG, the ethyl ester form (DHAH-EE) was liquid at room temperature, and the obtained lyophilized NLCs were easy to handle. However, the NLCs prepared with DHAH-TG became a soggy and sticky powder at room temperature, and this was more difficult to handle after the lyophilization process. That is why the nanoformulations developed with DHAH-TG (C1–C4, Figure 4) were discarded to continue working.

NLCs formed with DHA and DHAH-EE showed similar pharmaceutical characteristics; however, as we probed in a previous in vivo study, DHA and DHAH functional lipids showed different biological activity [31]. Therefore, taking into account the remarkably beneficial effects of DHAH, we chose DHAH-EE nanoformulations to continue working. Among the different nanoformulations developed with DHAH-EE (D1–D4, Figure 4), we selected D3 (51.40nm ± 11.65 and 0.415 ± 0.082 for PDI values) since it was the formulation with the highest percentage of Ω-3 fatty acid incorporated into the lipid matrix of NLCs with the best resuspension characteristics, qualitatively determined. In order to confirm the therapeutic effect of DHAH-EE in the newly developed NLCs, we also chose Mygliol-based NLCs in the same proportion of solid:liquid lipid (A3, Figure 4), with a particle size of 75.02 ± 6.97 nm and 0.474 ± 0.023 PDI value, as the control formulation.

All the data regarding size, PDI and Z potential of all the developed nanoformulations have been summarized in Table 2. As seen, the size of NLC decreased when the percentage of liquid lipid was higher. Moreover, the Z potential of all nanoformulations was negative, which was reverted after CS and TAT coating (Table 3).

In the next step, the CS and TAT coating process was performed. In Table 2, we can see the results from CS-TAT-NLC-DHAH-EE nanoformulation, called DHAH-NLCs, and from Mygliol-based nanoparticles with CS and TAT, called Mygliol-NLCs.

**Figure 5:**
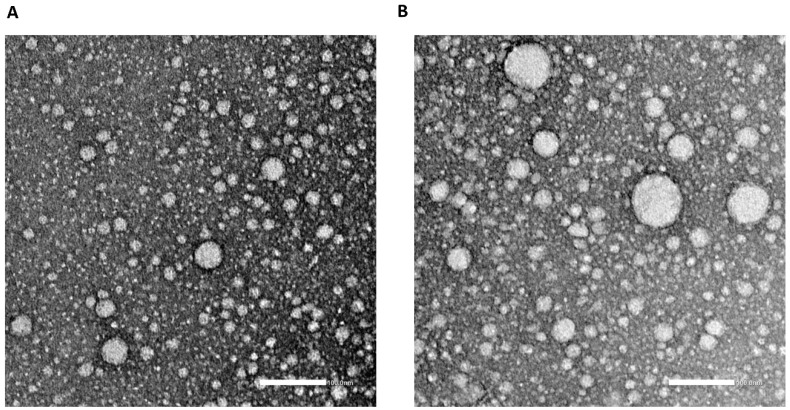
TEM (transmission electron microscopy) photographs of NLC (scale bar 100 nm). (**A**) hydroxylated derivate of docohexaenoic acid nanostructured lipids (DHAH-NLCs) (**B**) Mygliol-NLCs.

**Figure A1:**
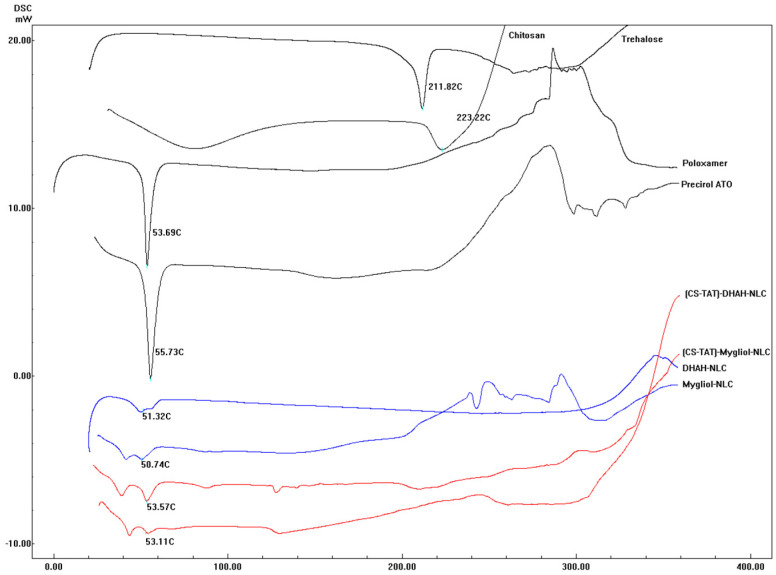
Differential scanning calorimetry (DSC) graphs of pure excipients (D-trehalose, Poloxamer 188, Precirol ATO 5, chitosan (CS) and the different developed nanoparticles).

Both formulations were around 100 nm in size, with a PDI value below 0.5, and exhibited positive zeta values, indicating that the CS and TAT coating process has been successfully performed. In the external morphological study made by TEM (transmission electron microscopy), the nanoparticles showed a uniform size without irregularities (Figure 5). The DSC thermograms of the different excipients and formulations have been summarized in Appendix Figure A1.

**Figure A2:**
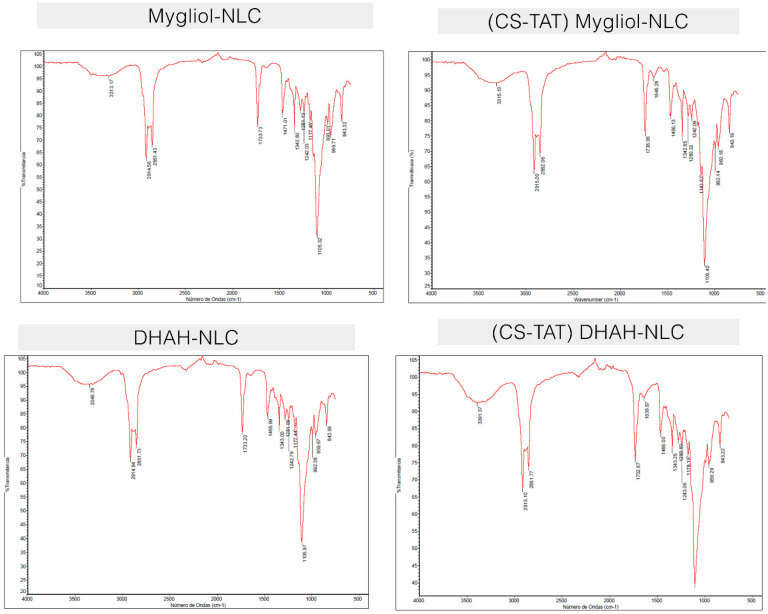
FTIR spectra of Mygliol-NLC, (CS-TAT) Mygliol-NLC, DHAH-NLC and (CS-TAT) DHAH-NL.

On the other hand, as seen in Figure A2, FTIR results exhibited a number of characteristic protein transmission bands (cm^−1^). Mygliol-NLC and DHAH-NLC without CS and TAT coating showed typical peaks of lipid components, such as O–H stretching (3315), aliphatic C–H (2915, CH3 and CH2) asymmetrical stretching, aliphatic C–H (2852, CH3 and CH2) symmetrical stretching and C=O (1736, carboxylic group) stretching in addition to the vibrations associated with C–O and C–C (1150 and from 992 to 843) bonds. Moreover, CH2 and CH3 stretching (1466) and bending (1342) bands can be seen. Regarding nanoformulations with TAT and CS coating (CS-TAT-Mygliol-NLC and CS-TAT-DHAH-NLC), a new peak can be seen, concretely, in amide I (N–H stretching) (1645). This new peak is due to a chemical interaction. Indeed, it is related to the presence of the amide bond formed after TAT peptide conjugation through a cross linking reaction.

### 3.2. Cell Cultures

**Figure 6:**
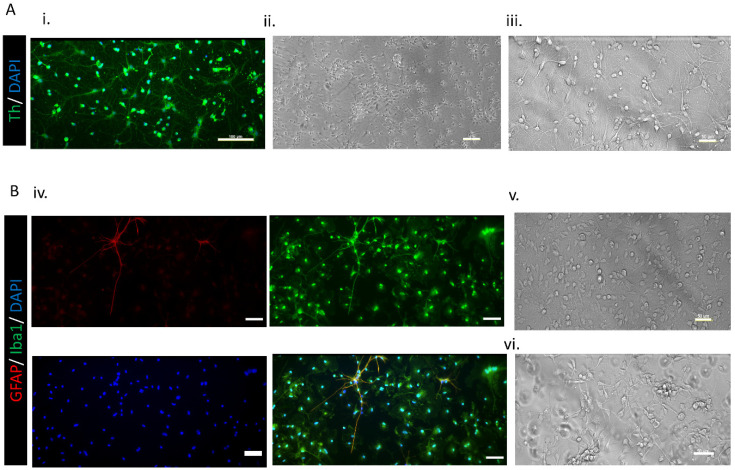
(**A**) Images of the primary dopaminergic cell culture. i: Immunofluorescence staining: positive for TH dopaminergic marker. (Scale bar 100 μM) ii: (scale bar 100 μM) and iii: (scale bar 50 μM) Bright field images of primary dopaminergic cell cultures. (**B**) Images of the primary microglia cell culture. Iv: Immunofluorescence staining. (Scale bar 50 μM) v: Bright field images of glia mix culture. (Scale bar 50 μM). vi: Bright field images of the primary microglia cell culture after isolation. (Scale bar 50 μM).

In order to assess the purity of our primary cultures, an immunofluoresce technique was performed in both dopaminergic and microglia cell cultures. The experiments were performed in triplicate and repeated at least three times independently (*n* = 3 biological replicates). Dopaminergic cell cultures were positive for TH dopaminergic marker, as shown in Figure 6A. In the case of the microglia cell culture, we tested both the microglia marker Iba1 and the astroglia maker GFAP. The culture was shown to be specific in a high percentage to microglia marker (Figure 6B. iv). However, it must be noted that, after this isolation method, the culture is not 100% specific for microglia cells, and also some astrocytes can be seen in a non-noteworthy way.

### 3.3. In Vitro Cell Viability Study

**Figure 7:**
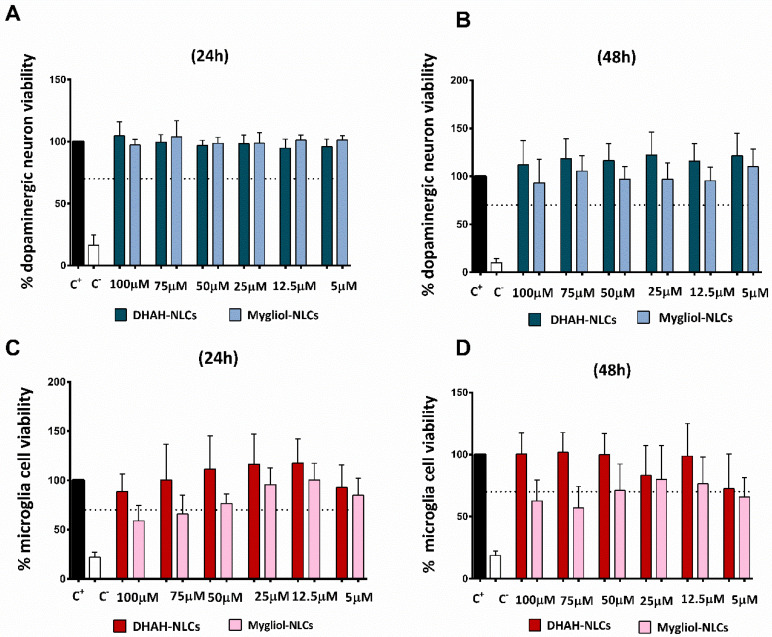
(**A**) Dopaminergic neuron viability at 24 h. (**B**) Dopaminergic neuron viability at 48 h. (**C**) Microglia cell viability at 24 h. (**D**) Microglia cell viability at 48 h. In all cases, different concentrations of DHAH-NLCs and Mygliol-NLCs were tested (the graphs show the results of three biological replicates media ± SD).

In order to assess the cytocompatibility of the nanoparticles, they were incubated with the dopaminergic and microglia cell cultures. After 24 and 48 h, cell viability was measured through the CCK-8 assay. Figure 7A,B illustrates the results obtained in the CCK-8 assay after incubating NLCs with a dopaminergic cell culture. None of the concentrations for the nanoformulations tested in dopaminergic cell cultures were cytotoxic, showing percentages of cell viability >70%. Indeed, as shown in Figure 7B, at 48 h, cell viability for the cultures treated with DHAH-NLCs was slightly better at any of the tested concentrations compared to Mygliol-NLCs treated cells. The results of the viability assay for the microglia cell culture are, to some extent, different. As shown in Figure 7C,D, the tested concentrations of 100 and 75 μM for the Mygliol-NLC formulation decreased cell viability below 70% at both tested time points; this effect was not seen with DHAH-NLC for any of the tested time points. This beneficial effect for DHAH-NLC was seen in any of the tested conditions, being more notorious at 50, 25 and 12.5 μM concentrations (Figure 7C,D).

### 3.4. 6-OHDA Neurotoxin Effect on Dopaminergic Culture

The incubation of dopaminergic neurons with 6-OHDA decreased cell viability in a dose-dependent manner (Figure 2B). The incubation of cells with 500 μM of 6-OHDA resulted in only 6.78 ± 4.11 (**** *p* < 0.0001) remaining living cells, which is similar to the values obtained after the incubation with our C^−^ (DMSO 10%) (5.45 ± 5.2, **** *p* < 0.0001). In the following test concentrations, the values for 100 and 50 μM were below 50% of the remaining living cells; more specifically, for 100 μM (13.82 ± 8.24, **** *p* < 0.0001) and for 50 μM (33.37 ± 11.87, **** *p* < 0.0001), the values were obtained. In the case of 25 μM, the remaining living cells were around 50% (49.05 ± 7.79, **** *p* < 0.0001), and, for 10 μM, it was slightly higher (60.37 ± 24.33, *** *p* < 0.001). Finally, the incubation of dopaminergic cells with 5 μM 6-OHDA neurotoxin led to no statistical difference when compared to the C^+^ group, with 87.17 ± 18.02 remaining living cells.

### 3.5. DHAH-NLCs Exhibited A Neuroprotective Effect

After selecting the dose for generating a cell death of 50% after 24 h of incubation with 6-OHDA, 25 μM, we tried to demonstrate the neuroprotective effect of our DHAH-NLCs. The incubation of the dopaminergic neuron culture with DHAH-NLCs before and after 6-OHDA addition resulted in a neuroprotective effect in comparison to the neurotoxin itself (Figure 2C). The treatment with Mygliol-NLCs did not increase cell viability, with remaining living cell values similar to those obtained with just the neurotoxin incubation. The values for 50 μM Mygliol NLCs (38.35 ± 33.45), 25 μM Mygliol NLCs (49.49 ± 23.15) and 12.5 Mygliol NLCs (54.13 ± 19.11) were similar, without any neuroprotective effect. In contrast, for those cells treated with DHAH-NLCs, the values for the remaining living cells were similar to the control set as 100%. There were no statistical differences between the tested doses with the following values: 50 μM DHAH-NLCs (84.22 ± 10.58, ** *p* < 0.01), 25 μM DHAH-NLCs (82.70 ± 18.96, ** *p* < 0.01) and 12.5 μM DHAH-NLCs (79.92 ± 17.06, * *p* < 0.05). The images taken with fluorescence microscopy showed the difference in living cells, comparing the ones treated with DHAH-NLCs with those treated Mygliol-NLCs (Figure 2D).

### 3.6. DHAH-NLCs Decreased Cytokine Proinflammatory Release

In order to assess the antiinflammatory effect of our DHAH-enriched nanoparticles (DHAH-NLCs), we performed the ELISA technique from a cell culture supernatant, as described in the Materials and Methods section. The results obtained from the cell supernatants for TNF-α, IL-1β and IL-6 cytokines are summarized in Figure 3B–D.

Regarding TNF-α ELISA (Figure 2B), the basal levels of TNF-α, C^−^ (204.0 ± 23.96) statically increased (**** *p* < 0.0001) after LPS treatment (50 ng/mL), named C^+^ (879.4 ± 204.0). In addition, although DHAH-NLCs were not able to obtain control levels of this cytokine, it is noteworthy that the effect of this treatment decreases this proinflammatory cytokine release, as seen in Figure 3B. This effect can be seen in all DHAH-NLCs concentrations: 50 μM DHAH-NLCs (581.0 ± 100.1, *** *p* < 0.001), 25 μM DHAH-NLCs (532.1 ± 186.0, **** *p* < 0.0001) and 12.5 μM DHAH-NLCs (609.2 ± 55.1, ** *p* < 0.01), without any statistically significant differences between the different doses. In the case of Mygliol-NLCs, we could not see any positive effect decreasing cytokine levels for any of the tested concentrations (*p* > 0.05).

For the second tested cytokine, IL-6 (Figure 3C), we also observed the increase of basal levels (213.7 ± 47.3) up to almost double concentration (365.1 ± 125.9, *** *p* < 0.001) after the incubation with LPS. In this case, we could also see the decrease of this proinflammatory cytokine after the treatment with DHAH-NLCs. All the tested concentrations demonstrated a positive effect, decreasing IL-6 levels; indeed, 50 μM DHAH-NLCs (219.3 ± 29.3, ** *p* < 0.01), 25 μM DHAH-NLCs (232.7 ± 40.8, * *p* < 0.05) and 12.5 μM DHAH-NLCs (237.2 ± 15.4, * *p* < 0.05) were the obtained values. However, no statistically significant differences were observed between the different concentrations. Regarding Mygliol-NLCs, no antiinflammatory effect was obtained after the treatment with these nanoparticles for any of the tested concentrations (*p* > 0.05).

Finally, we analyzed the levels of IL-1β proinflammatory cytokine (Figure 3D). In this case, the LPS stimuli also increased the basal values (457.4 ± 125.8) more than three-fold, as we can see for the C^+^ values (1495.0 ± 518.1, ** *p* < 0.01). As seen before with the other two proinflammatory cytokines, the treatment with DHAH-NLCs decreased IL-1β values. Actually, the values for DHAH-NLCs were the following: 50 μM DHAH-NLCs (726.2 ± 256.6, * *p* < 0.05), 25 μM DHAH-NLCs (948.7 ± 301.6) and 12.5 μM DHAH-NLCs (985.6 ± 301.0). Although in this case only the group treated with 50 μM DHAH-NLCs showed a statistically significant difference, the trend to decrease this proinflammatory cytokine is notorious in all DHAH-NLCs concentrations. As in the previously analyzed proinflammatory cytokines, the levels of IL-1β remained elevated for the cells treated with Mygliol-NLCs, for any of the tested conditions, showing values similar to the positive control.

## 4. Discussion

NDs are one of the main problems of the public health system in the 21st century. Although they have different clinical manifestations and symptoms, they all share a complex mechanism of neurodegeneration, with aging as the main risk factor, and they are without an effective treatment [1,48]. In order to obtain an effective treatment for NDs, in the last few years, different therapeutic molecules have been raised as new clinical candidates. Among others, the use of functional lipids such as PUFAs and, more concretely, DHA and DHAH have been raised as a useful tool to treat NDs since they exhibit beneficial effects, decreasing neuroinflammation and protein deposit or increasing the release of neuroprotective agents [30,49]. However, no matter the treatment, one of the main issues with treating NDs is reaching the brain and passing through the BBB. That is why the use of nanotechnology and, more concretely, the NLCs, has exhibited promising results for targeting the brain, increasing bioavailability, protecting from oxidation and, therefore, maintaining the bioactivity of different molecules [19].

The main goal of this work was to combine both strategies to develop a new therapeutic nanocarrier enriched with PUFAs, more concretely, with DHAH, to generate a new type of NP that could target the brain and exhibit neuroprotective and antiinflammatory effects, therefore becoming a functional nanocarrier that could be combined with different molecules in the future to promote a synergistic therapy.

All the developed nanoformulations were 50–90 nm in size, with a PDI value below 0.5 indicating a homogenous suspension (Figure 4). The increment of the liquid lipid ratio led to a decrease in the size of the nanoparticles, as shown in previous publications (Table 2) [50,51]. Moreover, as seen in DSC thermograms (Figure A1), the addition of the liquid lipid to the solid lipid led to a slight reduction of the melting point of Precirol of 3–4 °C in all NLC thermograms, which is similar to previously conducted studies [50,52,53]. Anyway, the selected solid:liquid lipid ratio for the two different lipids, Mygliol and DHAH, was in the ratio normally used for NLC preparation [24]. After selecting the ratio and the lipid to constitute the new nanoformulation, the NPs’ surface was modified with the addition of CS and TAT, resulting in two different formulations, named DHAH-NLCs and Mygliol-NLCs. The addition of CS and TAT peptide led to the conversion of Z potential from negative values to positive values (Table 2 and Table 3), indicating that the undergoing process was successfully performed. Moreover, the presence of amide I in FTIR spectra (Figure A2) confirmed that the coating process with TAT was successfully performed. As we have previously demonstrated, the addition of CS and TAT increased in vivo brain targeting after intranasal administration [16,24]. Moreover, TAT is a well-known CPP, usually used to enhance the delivery of different cargos into different types of cells, such as neurons [54,55]. The resulting NPs were similar in size (around 100 nm) and exhibited positive zeta values due to the successful CS coating process (Table 3).

Among the different NDs, in this research paper, we aim to study the effect of our newly developed nanocarriers in a cell model of PD by adding 6-OHDA neurotoxin, one of the most widely used neurotoxins, to a dopaminergic cell culture to mimic the destruction of catecholaminergic neurons and the degeneration of the nigrostriatal pathway [56]. The addition of 25 μM 6-0HDA for 24 h led to a decrease in cell viability of 50%, as shown in Figure 2B. The dose and incubation time for this neurotoxin is variable, according to the scientific data available, varying from 5 to 100 μM and from 30 min to 48 h [57,58,59,60]. That is why we incubated the neuronal cells with different doses for 24 h, an intermediate time point in the published articles, and selected the dose that generated 50% cell death so we could really see the effect of the neurotoxin on cell viability. In order to check the safety and effectiveness of the DHAH incorporated into our NLCs, we incubated the nanoformulations for 24 and 48 h. All tested concentrations were shown to be safe (Figure 7A,B), with a viability up to 70% and showing better values for DHAH-NLCs at 48 h. Moreover, the effectiveness of DHAH-NLCs was also evaluated in dopaminergic cell cultures after the incubation with 6-OHDA. DHAH-NLCs were shown to be effective at protecting the cells from the neurotoxin compared to Mygliol-NLCs as the control group (Figure 2C,D). These data are in line with previous publications regarding the DHA neuroprotective effect shown in neuron cell cultures [61,62]. Moreover, it demonstrates the effectiveness of the DHAH functional lipid incorporated into the newly developed NLCs.

On the other hand, the neuroinflammatory process undergoing NDs and, more specifically, in PD is well known, being the consequence or the cause of the disease. Whatever the origin of the neuroinflammation, it is a fact that a therapeutic intervention downregulating this process could be great at halting the progression of the disease [63]. Although different cell types and molecules are involved in the neuroinflammation, microglia cells are the primary initiators of the central inflammatory response to acute and chronic disorders related to NDs [64]. In order to treat this inflammatory response, PUFAs and, more concretely, DHA and DHAH have been raised as emerging candidates to downregulate this process and become a new therapeutic approach [9,31]. The DHAH-NLCs developed in this study were shown to be safe at any of the tested concentrations, with a viability up to 70%, in contrast to Mygliol-NLCs, where higher concentrations decreased cell viability (Figure 7C,D). Thus, these results demonstrated that the newly developed formulation with DHAH can be used in high concentrations without affecting cell viability.

In order to mimic the neuroinflammatory process in primary microglia cells, different molecules can be used. Among them, the gold standard stimuli for generating reactive gliosis and activating the neuroinflammation cascade is LPS. LPS has been widely used to generate animal models of neuroinflammation or induce it in cell culture, both primary and microglia cell lines [65,66,67]. The addition of LPS at 50 ng/mL concentration in this study generated the production of proinflammatory cytokines in similar levels to those in previously published scientific articles for microglia primary cell cultures isolated after the shaking method [66]. Regarding the potential antiinflammatory effect of our NPs, we showed that the ability of DHAH to decrease neuroinflammation was maintained in DHAH-NLCs, obtaining proinflammatory cytokine levels similar to C^−^ in IL-6 and IL-1β, and decreasing TNF-α almost by half for any of the tested concentrations (Figure 3B–D). The ability of PUFAs to decrease the cytokine proinflammatory levels has previously been described [68]; thus, this study enforces the use of these kinds of PUFAs formulated in NLCs as an emerging tool to treat the undergoing inflammatory process in NDs.

## 5. Conclusions

Altogether, these results highlight that the newly developed nanocarriers can constitute a new therapeutic tool for treating NDs. The DHAH-NLCs were similar to previously developed NLCs in size, PDI, zeta values and TEM morphology. Moreover, they exhibited neuroprotective effects in a cell culture model of dopaminergic neurons and antiinflammatory properties, decreasing proinflammatory cytokine levels in primary microglia cell cultures. Although future studies are needed to check its suitability in a different cell culture model or animal model of the disease, the results presented in this research article are promising for this new functional nanocarrier. Moreover, the combination of this new, safe and effective nanocarrier with different clinically approved or investigated therapeutic molecules could become in an emerging tool to treat, in a synergistic manner, the symptoms associated with NDs.

## Figures and Tables

**Table 1:** The composition of the different nanoparticles, using Precirol^®^ ATO 5 and different Ω-3 fatty acids or Mygliol^®^ as a liquid lipid with different solid:liquid lipid ratios.

Liquid Lipid	Formulation	% Precirol ATO 5^®^ (*w*/*v*)	% Liquid Lipid (*w*/*v*)	% T80 (*w*/*v*)	% Poloxamer 188 (*w*/*v*)
Mygliol^®^	A1	2.5	0.25	3	2
	A2	2	0.75	3	2
	A3	1.75	1	3	2
	A4	1.5	1.25	3	2
DHA	B1	2.5	0.25	3	2
	B2	2	0.75	3	2
	B3	1.75	1	3	2
	B4	1.5	1.25	3	2
DHAH-EE	C1	2.5	0.25	3	2
	C2	2	0.75	3	2
	C3	1.75	1	3	2
	C4	1.5	1.25	3	2
DHAH-TG	D1	2.5	0.25	3	2
	D2	2	0.75	3	2
	D3	1.75	1	3	2
	D4	1.5	1.25	3	2

**Table 2:** Characterization of the developed NLCs (*n* = 3 for all the nanoformulations). The data are presented in media ± SD.

Formulation	Size after Lyoph (nm)	PDI	Z Potential (mV)
Formulation A1	83.07 ± 36.54	0.342 ± 0.061	−15.6 ± 1.7
Formulation A2	73.11 ± 19.51	0.364 ± 0.071	−16.0 ± 2.8
Formulation A3	75.02 ± 6.97	0.474 ± 0.023	−14.2 ± 19.2
Formulation A4	59.38 ± 25.39	0.468 ± 0.052	−14.4 ± 4.8
Formulation B1	94.19 ± 18.01	0.350 ± 0.092	−19.4 ± 1.8
Formulation B2	85.20 ± 12.20	0.461 ± 0.174	−19.9 ± 3.1
Formulation B3	51.98 ± 10.70	0.420 ± 0.202	−22.7 ± 3.5
Formulation B4	49.93 ± 5.36	0.587 ± 0.118	−22.8 ± 4.1
Formulation C1	63.34 ± 6.95	0.366 ± 0.073	−21.0 ± 5.8
Formulation C2	67.02 ± 4.10	0.463 ± 0.031	−16.5 ± 2.3
Formulation C3	78.63 ± 24.66	0.467 ± 0.062	−20.1 ± 3.4
Formulation C4	80.87 ± 15.15	0.418 ± 0.056	−20.4 ± 1.5
Formulation D1	68.62 ± 16.70	0.404 ± 0.068	−22.7 ± 2.6
Formulation D2	76.68 ± 20.12	0.425 ± 0.101	−24.2 ± 2.9
Formulation D3	51.40 ± 11.65	0.415 ± 0.082	−24.1 ± 2.9
Formulation D4	39.98 ± 10.39	0.401 ± 0.076	−24.9 ± 2.8

**Table 3:** Characterization of the final nanoformulations used for cell culture tests. (*n* = 2 independent experiments).

Formulation	Size after Lyoph (nm)	PDI	Z Potential (mV)
DHAH-NLC	97.80 ± 2.00	0.274 ± 1.26	12.63 ± 1.26
Mygliol-NLC	94.31 ± 0.43	0.454 ± 0.007	14.13 ± 0.21
